# Arbuscular Mycorrhizal Fungi Alleviate Drought Stress in C_3_ (*Leymus chinensis*) and C_4_ (*Hemarthria altissima*) Grasses via Altering Antioxidant Enzyme Activities and Photosynthesis

**DOI:** 10.3389/fpls.2019.00499

**Published:** 2019-04-30

**Authors:** Junqin Li, Bo Meng, Hua Chai, Xuechen Yang, Wenzheng Song, Shuixiu Li, Ao Lu, Tao Zhang, Wei Sun

**Affiliations:** Key Laboratory of Vegetation Ecology, Ministry of Education, Institute of Grassland Science, Northeast Normal University, Changchun, China

**Keywords:** AMF, climate change, drought resistance, C_3_ and C_4_ species, antioxidant enzyme activities, photosynthesis

## Abstract

As one of the most important limiting factors of grassland productivity, drought is predicted to increase in intensity and frequency. Greenhouse studies suggest that arbuscular mycorrhizal fungi (AMF) can improve plant drought resistance. However, whether AMF can improve plant drought resistance in field conditions and whether the effects of AMF on drought resistance differ among plants with different photosynthetic pathways remain unclear. To evaluate the effect of indigenous AMF on plant drought resistance, an *in situ* rainfall exclusion experiment was conducted in a temperate meadow in northeast China. The results showed that AMF significantly reduced the negative effects of drought on plant growth. On average, AMF enhanced plant biomass, photosynthetic rate (*A*), stomatal conductance (*g*_s_), intrinsic water use efficiency (iWUE), and superoxide dismutase (SOD) activity of the C_3_ species *Leymus chinensis* by 58, 63, 38, 15, and 45%, respectively, and reduced levels of malondialdehyde (MDA) by 32% under light and moderate drought (rainfall exclusion of 30 and 50%, respectively). However, under extreme drought (rainfall exclusion of 70%), AMF elevated only aboveground biomass and catalase (CAT) activities. Averagely, AMF increased the aboveground biomass, *A*, and CAT activity of *Hemarthria altissima* (C_4_) by 37, 28, and 30%, respectively, under light and moderate droughts. The contribution of AMF to plant drought resistance was higher for the C_3_ species than that for the C_4_ species under both light and moderate drought conditions. The results highlight potential photosynthetic type differences in the magnitude of AMF-associated enhancement in plant drought resistance. Therefore, AMF may determine plant community structure under future climate change scenarios by affecting the drought resistance of different plant functional groups.

## Introduction

With the intensification of global climate change, drought has been becoming a ubiquitous global environmental problem (Piao et al., 2010; Trenberth et al., 2014; Mathur et al., 2018). Drought significantly suppresses plant growth and decreases net primary productivity in arid and semiarid grasslands (Shukla et al., 2012; Li et al., 2015). To alleviate the negative influences of drought on plant growth, plants are required to respond quickly by altering their morphological, physiological, and biochemical characteristics (Bardgett et al., 2014). However, the response of plant growth to drought stress is often regulated by soil microbes, such as mycorrhizal fungi. To date, the effects of soil microbes on plant drought resistance are not fully understood.

Arbuscular mycorrhizal fungi (AMF) are one of the most important groups of soil microbes and function as mycorrhizal symbionts with the roots of approximately 72% of terrestrial plants (Brundrett and Tedersoo, 2018). Many studies have demonstrated that AMF can improve the growth of host plants by promoting nutrient and water uptake to alleviate abiotic stresses, such as drought (Baum et al., 2015; Zhao et al., 2015; Bowles et al., 2018). AM fungal hyphae can explore soil pores that the root hair cannot contact, accessing water and nutrient sources that are not available to non-AM plants. Therefore, AMF can improve plant performance, change the plant–water relationship, and increase plant productivity under drought stress (Augé, 2001). Recent studies have proposed different mechanisms through which AMF alleviate drought, salt, or temperature stress. For instance, AMF can increase water use efficiency (WUE) by improving stomatal conductance (*g*_s_) (Augé et al., 2015) and increase antioxidant enzyme activity to reduce peroxidative damage (Pedranzani et al., 2016; Chang et al., 2018; Duc et al., 2018). In addition, Pedranzani et al. (2016) reported that AMF regulated plant physiological performance of *Digitaria eriantha* to alleviate drought, salinity, and cold stresses by the upregulation of antioxidant enzyme activity and jasmonate synthesis. Nevertheless, most previous studies on this topic were carried out under greenhouse conditions and the influences of AMF on plant drought resistance under field conditions are not well understood.

The contribution of AMF to drought resistance may differ among plant functional groups (e.g., C_3_ vs. C_4_ species; Yamori et al., 2014). Many studies have found that under drought conditions, the contribution of AMF to WUE was higher in C_3_ species than in C_4_ species (Edwards et al., 2010; Worchel et al., 2013; Augé et al., 2015). Furthermore, the degrees to which the activities of antioxidant enzymes and the amount of antioxidants are elevated under drought stress show pronounced variation among plant species (Türkan et al., 2005). C_4_ plants are reported to be better adapted to water stress than are C_3_ plants due to multiple physiological mechanisms specific to C_4_ plants (Pearcy and Ehleringer, 1984; Nelson et al., 2004). Under drought conditions, the antioxidant defense system in C_3_ plants has been found insufficient to suppress the increase in reactive oxygen species (ROS) production induced by drought (Uzilday et al., 2012). Therefore, under drought stress, C_3_ species are expected to face more severe oxidative damage than are C_4_ species. C_3_ species may use other external means (e.g., symbiosis with AMF) to improve their antioxidant defense systems under drought conditions. However, the influences of AMF on plant drought resistance in plant species with different photosynthetic pathways remain unclear.

The aims of this study were to investigate the effects of AMF on the drought resistance of C_3_ (*Leymus chinensis*) and C_4_ (*Hemarthria altissima*) species in a temperate meadow ecosystem. An *in situ* rainfall reduction experiment (involving the exclusion of growing-season rainfall by 0, 30, 50, or 70%) was conducted in the Songnen grassland in northeastern China. We hypothesized that (1) AMF would improve plant growth by affecting photosynthetic rate, WUE, and antioxidant enzyme activity under light and medium drought stress; (2) AMF would not affect plant growth under the extreme drought treatment; and (3) the contribution of mycorrhizae to drought resistance would be higher in the C_3_ grass *L. chinensis* than in the C_4_ grass *H. altissima*.

## Materials and Methods

### Ethics Statement

No specific permissions were required for the field studies described, because the Songnen Grassland Ecological Research Station is a department of the Northeast Normal University. No specific permissions were required for the study either, as it was conducted in accordance with the guidelines set by the Northeast Normal University. No specific permissions were required for the locations or the activities. No location was privately owned or protected in any way, and the field studies did not involve endangered or protected species.

### Experimental Site

The experiment was conducted in the Songnen meadow, which is located in western Jilin Province, northeast China (44°40′–44°44′ N, 123°44′–123°47′ E). The study area has a temperate semiarid continental climate with an annual mean temperature ranging from 4.6 to 6.4°C (1950–2004), and an annual precipitation of 280–644 mm (1950–2014), with more than 70% of the precipitation concentrated in the summer (from June to August). The precipitation is only one-third of the potential evapotranspiration. The studied temperate meadow is dominated by the perennial grass *L. chinensis* (C_3_); other perennial grasses, such as *H. altissima* (C_4_) and *Phragmites australis* (C_4_) are abundant (Wang et al., 2018). In the studied grassland, the dominant taxon of arbuscular mycorrhiza is the genus *Glomus* spp. (Zhang et al., 2016). The soil is classified as chernozem and has a soil organic carbon content of 2.0% and a soil total nitrogen content of 0.15% (Zhong et al., 2017; Shi et al., 2019).

### Design of the In-Growth Core System

A modified in-growth core system was used for isolation of the root and AM fungal mycelial growth zone (Johnson et al., 2001; Zhang et al., 2011). Cores were constructed using PVC (polyvinyl chloride) tubes (height, 20 cm; inner diameter, 5 cm). Each core had two rectangular “windows” (each 10 cm in length and 4 cm in width), which together were equivalent to approximately 50% of the below ground external surface area. Nylon mesh (pore size of 35 μm) was glued to the cores to cover the “windows” and the base of the core to allow AM fungal hyphae but not roots to pass through. The bottoms of cores were sealed to prevent the growth of both roots and AM fungal hyphae beyond the bottoms of the cores.

### Design of the Rainout Shelter

The rainout shelters used for the present study were designed according to the method described by Yahdjian and Sala (2002). Each rainout shelter consisted of a metal frame and V-shaped clear acrylic bands (3.7 m long, 0.33 m wide, 3 mm thick, and arranged in a longitudinal plait of 120°). The type of acrylic material that was used only intercepts a small portion of direct solar radiation (<10%), and its elasticity is sufficient to withstand the gale conditions of our research site. The roof of the shelter had a 10° inclination, and the mean height of the shelter was 1.5 m (1.3 m on the lower side and 1.7 m on the higher side).

### Soil and Plant Preparation

The meadow steppe soil used to fill the cores were sieved (2-mm sieve) to remove large rocks, plant roots, and other litters, and then sterilized at 121°C for 2 h. The cores filled with sterilized soil were watered with deionized water. Seedlings of *L. chinensis* (C_3_) and *H. altissima* (C_4_) were dug from the studied grassland with a shovel and transported to the laboratory with the soil. To remove the AMF from the soil, the seedling roots were soaked in benomyl solution (9 g active benomyl in 15 L water) for 15 min (O’Connor et al., 2002). The AMF-free seedlings were then transplanted into the in-growth cores. The transplanted seedlings were allowed to establish for 2 weeks (and watered with deionized water) in the greenhouse before being placed in the field plots.

### Experimental Design

The experiment included two treatment factors: drought and AMF. There were four drought treatments comprising rainfall exclusion of 0% (RE0%, control), 30% (RE30%, light drought), 50% (RE50%, moderate drought), and 70% (RE70%, extreme drought), and they were replicated four times. Each drought treatment included two AMF treatments: static cores (AMF) and rotation cores (AMF-free). In 2015, we fenced a grassland area of 1 ha (100 × 100 m). Within the fenced area, four experimental blocks were established, each 25 × 25 m. There was at least 3 m between blocks. Four 3.5 × 3.5 m plots were established in each block, with at least 2 m between the plots. The four plots were randomly assigned to the RE0%, RE30%, RE50%, and RE70% treatments. The vegetation survey results indicated that there were no significant differences among treatments in species composition and aboveground biomass (data not shown). The drought treatments were initiated in April 2016 by using the rainout shelters for rainfall exclusion. Four, six, or eight bands of acrylic were mounted equidistantly in the roof of each rainout shelter to achieve the passive exclusion of 30, 50, or 70%, respectively, of the rainfall. For the plots subjected to 0% rainfall exclusion, we installed rainout shelters (providing 30% rainfall exclusion) and used the intercepted rainfall to manually water the RE0% plots immediately after each rainfall event. To test the effects of the rainout shelter on plant photosynthesis, one control treatment (without a rainout shelter) was established. The results showed that there was no difference in net assimilation rate (*A*) or stomatal conductance (*g*_s_) of either species between the RE0% and control treatments (Supplementary Figure S1).

From 2016 to 2018, rainfall exclusion treatments were conducted throughout the entire growing season (mid-April to mid-October). Water-blocking plates (stainless steel: 0.5 m belowground and 0.15 m aboveground) were placed around each plot to avoid water from overland runoff entering the plots and to avoid belowground lateral soil infiltration. On 14th May 2018, four cores of each of the C_3_ species (*L. chinensis*) and the C_4_ species (*H. altissima*) were randomly installed in each plot (0.5 m from the water-blocking plate). Half of the cores were rotated approximately 45° around their vertical axes every 2 days to break any hyphae penetrating into the cores (Rotation). The remaining half were left in place to allow the penetration of hyphae into the cores (Static).

### Air Temperature, Precipitation, and Soil Water Content

Climate data for the entire growing season of 2018, including precipitation and air temperature, were measured using an RG2-M sensor (Onset Computer Corporation, Bourne, MA, United States). Soil water content (0–10 cm) was measured once per month by oven drying 100 cm^-3^ soil samples collected from each plot during the experimental period (May to August in 2018).

### Measurements of Photosynthetic Characteristics

Leaf photosynthetic characteristics were measured using a portable photosynthetic apparatus (LI-6400, LI-COR Inc., Lincoln, NE, United States) between 07:30 and 11:00 on the day before in-growth core harvest (25th August). Leaf gas exchange was measured on the uppermost fully expanded leaves according to the method described by Chen et al. (2005). For each in-growth core, leaf gas exchange measurements were conducted on three leaves. Intrinsic WUE (iWUE) was determined as the ratio of net assimilation rate to stomatal conductance (*A*/*g*_s_).

### Plant Biomass and Mycorrhizal Colonization

The cores together with the plants were harvested on 25th August after 100 days of growth in the field. In each plot, one static core and one rotation core of each species were randomly selected for biomass assessment. Shoots, leaves, and roots were oven-dried at 70°C for 48 h and then weighed. Leaf water content was calculated and expressed on a fresh weight basis (1 - dry-weight/fresh-weight). The remaining two cores from each species were used for sampling of fresh plant materials. The sampled leaves were frozen in liquid nitrogen and stored at -80°C for subsequent measurements of enzyme activities.

Root colonization was measured by the visual observation of fungal structures under a microscope (BA210, Motic China Group Co., Ltd.) after washing with 10% (w/v) KOH and staining with 0.05% trypan blue in lactic acid (v/v; Phillips and Hayman, 1970). The quantification of root colonization (F%) and the intensity of mycorrhizal colonization (M%) were estimated according to the method described by Trouvelot et al. (1986).

### Lipid Peroxidation Level

The level of lipid peroxidation in leaf tissue was expressed as the amount of malondialdehyde (MDA) produced in a thiobarbituric acid reaction. The concentration of MDA was assessed according to the method described by Heath and Packer (1968). In brief, 0.3 g of fresh leaf sample was homogenized in 5 ml of 50 mM sodium phosphate buffer (pH 7.8). The homogenate was centrifuged at 10,000 *g* for 10 min. Four milliliters of 0.5% thiobarbituric acid containing 5% trichloroacetic acid was added to the supernatant. The mixture was heated at 95°C for 30 min and then quickly cooled in an ice bath. Next, the tube was centrifuged at 10,000 *g* for 10 min, and the absorbance of the supernatant at 532 and 600 nm was read. Each sample was analyzed three times.

### Antioxidant Enzyme Activities

Fresh leaf samples (0.5 g) were homogenized with a pestle in an ice-cold mortar containing 6 ml of ice-cold 50 mM sodium phosphate buffer (pH 7.0) containing 0.2 mM EDTA and 1% (w/v) polyvinylpyrrolidone. The homogenate was filtered through gauze and then centrifuged at 15,000 *g* for 20 min at 4°C, and the supernatant (enzyme extract) was collected and used for the measurement of superoxide dismutase (SOD), catalase (CAT), and POD activity (Zhang and Kirkham, 1994). Each sample was measured three times. Antioxidant enzyme activity was expressed on a protein basis, and the protein content of the enzyme extract was determined using bovine serum albumin (BSA) as a standard according to the method described by Bradford (1976).

The activities of SOD, CAT, and POD were determined according to the method described by Zhang and Kirkham (1996). For the measurements of total SOD activity, a 2.8-ml reaction mixture [containing 1.5 ml of 50 mM phosphate buffer (pH 7.8), 0.3 ml of 130 mM methionine, 0.3 ml of 750 μM NBT, 0.3 ml of 100 mM EDTA, 0.3 ml of 20 μM riboflavin, and 100 μl of enzyme extract] was placed under fluorescent lamps at 4,000 lx and 25°C for 20 min. The absorbance was then recorded with a spectrophotometer at 560 nm. A non-irradiated reaction mixture was used as a control. One unit of SOD was defined as the amount of enzyme required to inhibit the reduction rate of NBT by 50% at 25°C. For the measurement of CAT activity, a 3-ml reaction mixture containing 1 ml of 50 mM phosphate buffer (pH 7.0), 1.7 ml of deionized water, 0.2 ml of 15 mM H_2_O_2_, and 0.1 ml of enzyme extract was used. The absorbance at 240 nm was continuously measured for 3 min (25°C) after the addition of the enzyme extract and recorded every 30 s. Since the absorbance of hydrogen peroxide reached a maximum value at 240 nm, the CAT activity was expressed as the decomposition of H_2_O_2_ as measured by the decrease in absorbance at 240 nm for 3 min. For POD activity, a 2.98-ml reaction mixture containing 2.83 ml of 10 mM phosphate buffer (pH 7.0), 50 μl of 20 mM guaiacol, and 0.1 ml enzyme extract was used. POD activity was calculated by measuring the oxidation of guaiacol as determined by the change in absorbance at 470 nm over 1 min.

### Statistical Analysis

The mycorrhizal response (MR) to drought was calculated by the following formula using values of the static core and the mean values of the rotation cores for plant biomass, photosynthetic characteristics, MDA, and antioxidant enzyme activities in each rainfall exclusion treatment (Watts-Williams et al., 2013; Johnson et al., 2014; Bowles et al., 2018):

$$\%MR=\frac{\mathrm{value}\;(\mathrm{Static})\;-\;\mathrm{value}\;(\mathrm{Rotation})}{\mathrm{value}\;(\mathrm{Rotation})}$$

The effects of AMF, rainfall exclusion and their interaction on leaf gas exchange parameters, MDA, CAT, SOD, and POD activities were assessed by two-way analysis of variance (ANOVA). One-way ANOVA followed by Tukey’s *post hoc* comparisons were used to determine differences in soil water content, mycorrhizal colonization, and plant characteristics among the drought treatments and between the AMF treatments for each species. All statistical analyses were performed using SPSS 19.0 software (SPSS Inc., Chicago, IL, United States). Significant treatment effects were determined at *P* < 0.05. The results were expressed as the mean value ± standard error (SE, *n* = 4).

## Results

### Air Temperature, Precipitation, and Soil Water Content

**FIGURE 1:**
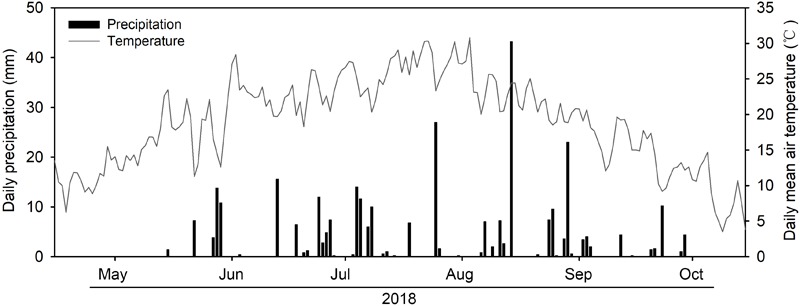
Daily precipitation (bars, mm) and daily mean air temperature (lines, °C) of growing season (May to October) in 2018.

**FIGURE 2:**
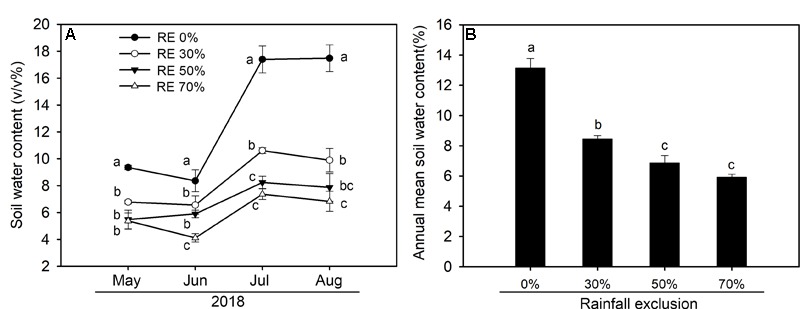
Seasonal dynamics of soil water content (V/V%) **(A)** and seasonal mean soil water content **(B)** at 0–10 cm depth of each rainfall exclusion treatment in 2018. RE0%, 0% rainfall exclusion; RE30%, 30% rainfall exclusion; RE50%, 50% rainfall exclusion; RE70%, 70% rainfall exclusion. Different lowercase letters indicate significant differences (*P* < 0.05) among the rainfall exclusion treatments. Data are reported as mean ± 1 SE (*n* = 4).

In 2018, the amount of growing season rainfall was 308.2 mm with over 60% of the rainfall occurring from July to August (Figure 1). There were pronounced temporal dynamics of daily mean air temperature during the growing season (Figure 1). Substantial seasonal variations in soil water content were observed due to variations in the timing and magnitude of rainfall events (Figure 2). We detected significant differences in mean soil water content between each combination under the four rainfall exclusion treatments, except for between RE50% and RE70% (Figure 2).

### Mycorrhizal Colonization

**FIGURE 3:**
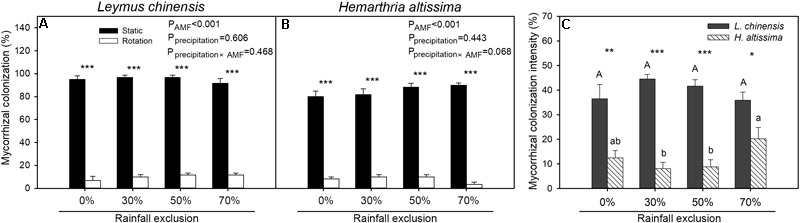
The percentage of arbuscular mycorrhizal fungi (AMF) roots colonization (F%) in *L. chinensis* **(A)** and *H. altissima* **(B)**, and the intensity of mycorrhizal colonization (M%) **(C)**. Different lowercase and capital letters indicate significant differences (*P* < 0.05) among the rainfall exclusion treatments in *H. altissima* and *L. chinensis*, respectively. ^∗∗∗^*P* < 0.001, ^∗∗^*P* < 0.01, and ^∗^*P* < 0.05 indicates differences between *L. chinensis* and *H. altissima*. Data are reported as mean ± 1 SE (*n* = 4).

In all of the rainfall exclusion treatments, mycorrhizal colonization in the root systems of *L. chinensis* and *H. altissima* was significantly higher (*P* < 0.05) for the static cores than for the rotation cores (Figure 3A,B). Mycorrhizal colonization intensity was significantly greater (*P* < 0.05) in the root of *L. chinensis* than in those of *H. altissima* across the four rainfall exclusion treatments (Figure 3C). For both species, there were no differences in intensity of mycorrhizal colonization between the RE0% treatment and any of the other rainfall exclusion conditions (RE30%, RE50%, and RE70%); however, in *H. altissima*, mycorrhizal colonization intensity in the RE70% treatment was higher than that in the RE30% or RE50% treatment.

### Plant Biomass

**FIGURE 4:**
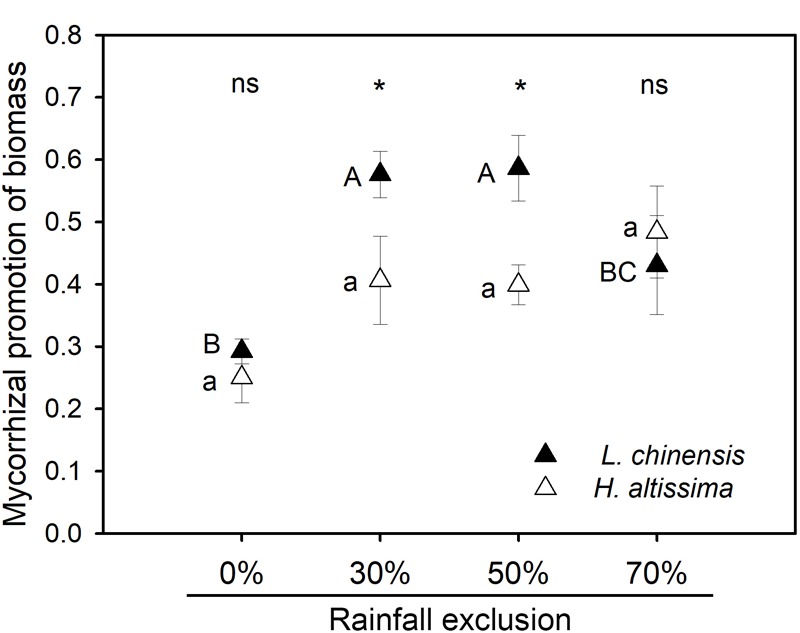
Mycorrhizal response of biomass in *L. chinensis* and *H. altissima*. Mycorrhizal benefit existed when values of mycorrhizal response were greater than zero. Different lowercase and capital letters indicate significant differences (*P* < 0.05) among the rainfall exclusion treatments in *H. altissima* and *L. chinensis*, respectively. ^∗^*P* < 0.05 indicates significant and ns (*P* > 0.05) indicates no differences between *L. chinensis* and *H. altissima*. Data are reported as mean ± 1 SE (*n* = 4).

For both species, rainfall exclusion significantly reduced plant biomass (*P* < 0.001, Table 1). Across the four rainfall exclusion treatments, AMF meanly increased plant biomass of *L. chinensis* and *H. altissima* by 47% (*P* = 0.003, Table 1, and Supplementary Figure S2A) and 37% (*P* = 0.011, Table 1, and Supplementary Figure S2B), respectively. AMF did not affect belowground biomass except in *L. chinensis* under the RE50% treatment. Significant AMF effects on the aboveground biomass of *L. chinensis* (*P* = 0.002) and *H. altissima* (*P* = 0.007) and belowground biomass of *L. chinensis* (*P* = 0.01) were observed (data not shown). For both species, no interactive effects between rainfall exclusion and AMF were detected on either aboveground or belowground biomass. For *L. chinensis*, the contributions of mycorrhizae on plant biomass in the RE30% and RE50% treatments were greater than the contribution in the RE0% treatment (Figure 4). However, in *H. altissima*, no difference in mycorrhizal contribution of plant biomass was observed among the four rainfall exclusion treatments (Figure 4). Interspecific differences in mycorrhizal promotion of biomass were detected in the RE30% and RE50% treatments (Figure 4 and Supplementary Table S1). For both species, there was no difference in leaf water content between the static and rotation treatments under any rainfall exclusion treatment (Supplementary Figure S3).

**Table 1:** Results of two-way ANOVA on the effects of rainfall exclusion (RE), arbuscular mycorrhizal fungi (AMF), and their interactions on biomass, photosynthesis characteristic, contents of malondialdehyde (MDA), and antioxidant enzyme activities in *L. chinensis* and *H. altissima*.

Source of variation	***L. chinensis***						***H. altissima***					
	**AMF**		**RE**		**RE × AMF**		**AMF**		**RE**		**RE × AMF**	
	***F***	***P***	***F***	***P***	***F***	***P***	***F***	***P***	***F***	***P***	***F***	***P***
Biomass	77	0.003	40	<0.001	1.5	0.270	32	0.011	33	<0.001	0.49	0.697
*A*	20	0.020	134	<0.001	10	0.003	17	0.025	71	<0.001	2.7	0.108
*g*_s_	18	0.023	45	<0.001	2.5	0.131	116	0.002	82	<0.001	1.1	0.406
iWUE	4.0	0.139	0.30	0.820	1.5	0.286	0.23	0.665	93	<0.001	0.47	0.711
MDA	35	0.010	15	0.001	5.9	0.017	0.038	0.858	110	<0.001	2.8	0.104
CAT	36	0.009	15	0.001	11	0.002	8.3	0.064	2.8	0.100	1.0	0.418
SOD	24	0.016	4.1	0.044	1.7	0.229	0.021	0.895	4.4	0.037	0.38	0.767
POD	32	0.011	0.81	0.519	0.23	0.874	0.053	0.833	0.61	0.624	0.085	0.967

*iWUE, intrinsic water use efficiency; CAT, catalase; SOD, superoxide dismutase.*

### Plant Photosynthesis Characteristics

The rainfall exclusion treatment had a significant effect on net assimilation rate (*A*) in both *L. chinensis* (*P* < 0.001, Table 1) and *H. altissima* (*P* = 0.001, Table 1). Relative to the rotation treatment, the static treatment enhanced *A* in *L. chinensis* by 25% (*P* < 0.05), 41% (*P* < 0.05), and 85% (*P* < 0.05) under the RE0%, RE30%, and RE50% treatments, respectively (Supplementary Figure S4A). For *H. altissima, A* in the static treatment was 14% (*P* < 0.05), 42% (*P* < 0.05), and 23% (*P* < 0.05) greater than that in the rotation treatment under the RE0%, RE30%, and RE50% conditions, respectively (Supplementary Figure S4B). For both species, rainfall exclusion and AMF had significant effects on *A* (*P* < 0.05, Table 1). A significant interactive effect between rainfall exclusion and AMF on *A* was detected only in *L. chinensis* (*P* = 0.003, Table 1).

In the *L. chinensis*, the static treatment enhanced stomatal conductance (*g*_s_) by 34% (*P* < 0.05), 27% (*P* < 0.05), and 50% (*P* < 0.05) under the RE0%, RE30%, and RE50% conditions, respectively (Supplementary Figure S4C). In *H. altissima*, the static treatment did not affect *g*_s_ except for increasing *g*_s_ by 39% (*P* < 0.05) in the RE50% treatment (Supplementary Figure S4D). Significant rainfall exclusion and AMF effects on *g*_s_ were observed in both species (*P* < 0.05, Table 1). There was no interactive effect between rainfall exclusion and AMF on *g*_s_ in either species (*P* > 0.05, Table 1).

Rainfall exclusion significantly affected iWUE in *H. altissima* (*P* < 0.001) but had no impact on iWUE in *L. chinensis* (*P* > 0.05, Table 1). The iWUE of *L. chinensis* in the static treatments were 12 and 19% higher than those in the rotation treatments under the RE30% and RE50% conditions, respectively (Supplementary Figure S4E). In *H. altissima*, no difference in iWUE was detected between the rotation and static treatments in any of the rainfall exclusion treatments (Supplementary Figure S4F). In *H. altissima*, significant effects of rainfall exclusion on iWUE were detected (*P* < 0.001, Table 1). No significant interactive effects between rainfall exclusion and AMF on iWUE were observed in either *L. chinensis* or *H. altissima*.

**FIGURE 5:**
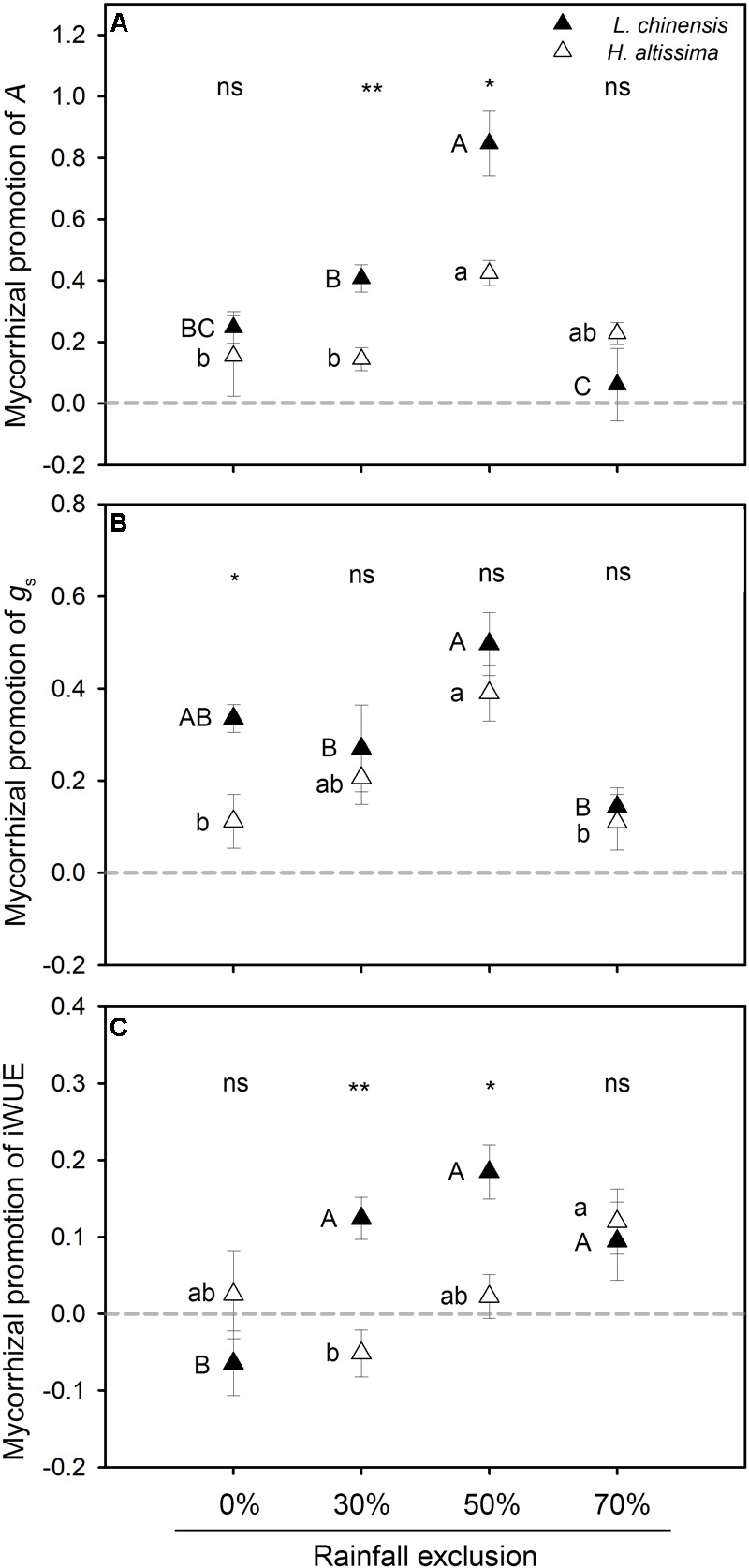
Mycorrhizal response of photosynthesis characteristic, including **(A)** net assimilation rate (*A*), **(B)** stomatal conductance (*g*_s_), and **(C)** intrinsic water use efficiency (iWUE) in *L. chinensis* and *H. altissima*. Mycorrhizal benefit existed when values of mycorrhizal response were greater than zero. Different lowercase and capital letters indicate significant differences (*P* < 0.05) among the rainfall exclusion treatments in *L. chinensis* and *H. altissima*, respectively. ^∗^*P* < 0.05 and ^∗∗^*P* < 0.01 indicates significant differences and ns (*P* > 0.05) indicates no differences between *L. chinensis* and *H. altissima*. Data are reported as mean ± 1 SE (*n* = 4).

In both species, the highest values of mycorrhizal contribution to *A* and *g*_s_ were observed under the RE50% treatment (Figure 5A,B). For *L. chinensis*, but not *H. altissima*, values of mycorrhizal promotion of iWUE were much higher under the RE30%, RE50%, and RE70% treatments than under the RE0% treatment. Moreover, the contributions of mycorrhizae to *A* and iWUE were significantly higher in *L. chinensis* than in *H. altissima* under the RE30% and RE50% treatments (Figure 5A,B). An interspecific difference in the contribution of mycorrhizae to *g*_s_ was detected only in the RE0% treatment (Figure 5B).

### Malondialdehyde

**FIGURE 6:**
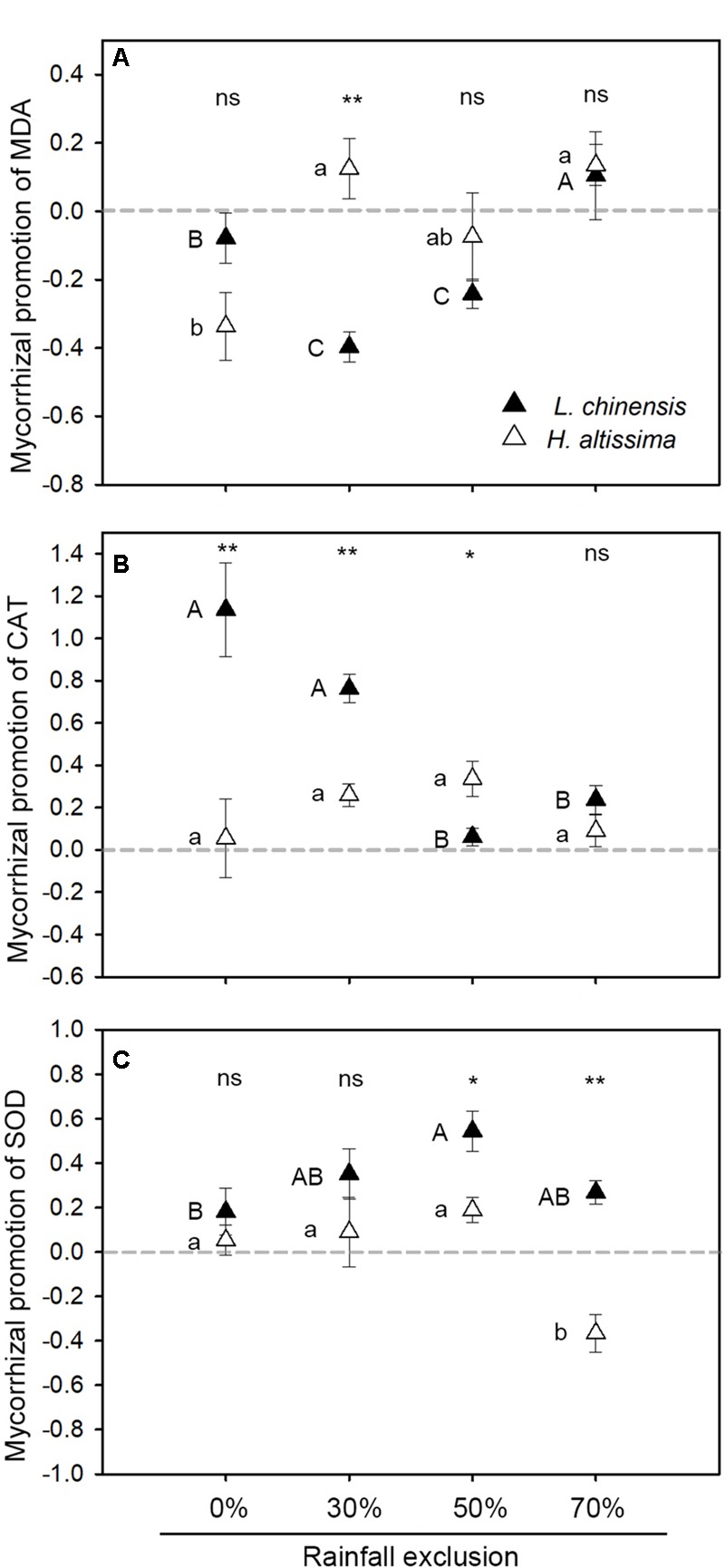
Mycorrhizal response of malondialdehyde (MDA, **A**) and antioxidant enzyme, including catalase (CAT, **B**), and superoxide dismutase (SOD, **C**), in *L. chinensis* and *H. altissima*. Mycorrhizal benefit existed when values of mycorrhizal response were greater than zero. ^∗^*P* < 0.05 and ^∗∗^*P* < 0.01 indicates significant differences and ns (*P* > 0.05) indicates no differences between *L. chinensis* and *H. altissima*. Data are reported as mean ± 1 SE (*n* = 4).

In *L. chinensis*, the static treatment reduced MDA content by 66 and 32% relative to that under the rotation treatment under the RE30% and RE50% conditions, respectively (Supplementary Figure S5A), whereas AMF treatment had no impact on MDA content under the RE0 and RE70% conditions. In *H. altissima*, there was no effect of AMF on MDA content in any of the rainfall exclusion treatments (*P* > 0.05, Table 1, Supplementary Figure S5B). In *L. chinensis*, MDA content was strongly influenced by the level of rainfall exclusion (*P* = 0.01), AMF (*P* = 0.001), and their interactions (*P* = 0.017, Table 1). In *H. altissima*, only rainfall exclusion treatment had significant effects on the content of MDA (*P* < 0.001, Table 1). In *L. chinensis*, relative to the mycorrhizal contribution to MDA in the RE0% treatment, the contribution was significantly reduced under the RE30% and RE50% treatments but enhanced under the RE70% treatment (Figure 6A). In *H. altissima*, the mycorrhizal contribution to MDA was significantly increased under the RE30% and RE70% treatments relative to that under the RE0% treatment, but no such increase was observed in the RE50% treatment. An interspecific difference in the mycorrhizal contribution to MDA content was detected only in the RE30% treatment.

### Antioxidant Enzyme Activities in Leaves

In *L. chinensis*, rainfall exclusion (*P* = 0.001), AMF (*P* = 0.009), and their interaction (*P* = 0.002) had significant effects on CAT activity (Table 1). In this species, relative to the rotation treatment, the static treatment caused 114% (*P* < 0.05), 76% (*P* < 0.05), and 35% (*P* < 0.05) increases in CAT activity under the RE0%, RE30%, and RE70% conditions, respectively, whereas there was no effect of AMF on CAT activity in the RE50% treatment (Supplementary Figure S6A). In *H. altissima*, under the RE30% and RE50% conditions, the static treatments enhanced CAT activity by 26% (*P* < 0.05) and 34% (*P* < 0.05), respectively (Supplementary Figure S6B). In *L. chinensis*, mycorrhizal promotion of CAT activity was significantly lower under the RE50% and RE70% treatments than under the RE0% treatment (Figure 6B). In *H. altissima*, no difference in mycorrhizal promotion of CAT activity was detected among the four rainfall exclusion treatments (Figure 6B). In addition, under the RE0% and RE30% conditions, the contribution of mycorrhizae to CAT activity was much higher in *L. chinensis* than in *H. altissima*, whereas the opposite pattern was observed for the ER50% treatment.

In both species, SOD activity was strongly affected by the rainfall exclusion treatment (*P* < 0.05, Table 1). AMF significantly affected SOD activity only in *L. chinensis* (*P* = 0.016, Table 1). Compared to the rotation treatment, the static treatment caused 35% (*P* < 0.05) and 54% (*P* < 0.05) increases in SOD activity in *L. chinensis* under the RE30% and RE50% conditions, respectively (Supplementary Figure S6C). In *H. altissima*, the static treatment increased (relative to the rotation treatment) SOD activity by 19% (*P* < 0.05) under the RE50% treatment but significantly reduced it by 37% (*P* < 0.05) under the RE70% treatment (Supplementary Figure S6D). In *L. chinensis*, the contribution of mycorrhizae to SOD activity was significantly higher under the RE50% treatment than under the RE0% treatment, whereas no difference was observed between the RE0% treatment and the RE30% or RE70% treatment (Figure 6C). In *H. altissima*, relative to the contribution of mycorrhizae to SOD activity in the RE0% treatment, this contribution was markedly reduced under the RE70% treatment (Figure 6C). Under the RE50 and RE70% conditions, the contributions of mycorrhizae to SOD activity were significantly greater in *L. chinensis* than in *H. altissima*.

In both species, there was no difference in POD activity between the static and rotation treatments in any of the four rainfall exclusion treatments (Supplementary Figures S6E,F).

## Discussion

### Effects of AMF Colonization on Plant Biomass

By intensifying the hydrological cycle, the ongoing global warming will increase drought frequency and magnitude, and these increases are likely to have profound impacts on plant growth and physiological performance, especially in water-limited ecosystems. In the present study, rainfall exclusion inhibited plant growth and the accumulation of aboveground biomass (Supplementary Figure S2), which is consistent with the results of previous studies (Fay et al., 2003; Muller et al., 2011; Xia et al., 2018). These results might be attributed to drought-associated reductions in leaf carbon assimilation rate.

It has been demonstrated that AMF can alleviate the negative effects of drought and improve plant growth under greenhouse conditions (Porcel and Ruiz-Lozano, 2004; Abbaspour et al., 2012; Bárzana et al., 2014). In the field conditions of the present study, relative to the rotation treatment, the static treatment (which allowed AMF colonization) significantly increased plant biomass, especially aboveground biomass (Figure 4; Supplementary Figure S2). This finding is in agreement with the results of previous studies demonstrating that AMF can effectively improve plant productivity under various water-deficit stresses (Gholamhoseini et al., 2013). The improvement of plant growth due to AMF might be explained by changes in both the photosynthesis and antioxidant capacities of plants. In the present study, AMF significantly enhanced leaf carbon assimilation rate and iWUE in *L. chinensis* under the RE30% and RE50% conditions (Supplementary Figure S4). Similar results have been reported in C_3_ crops under both well-watered and water-stress conditions (Wu and Xia, 2006; Ruiz-Sánchez et al., 2010; Zhang et al., 2018). In *L. chinensis*, the mycorrhizae contributions on *A* were much greater than its contribution on stomatal conductance under both the RE30% and RE50% conditions, which eventually caused increases in iWUE under these conditions (Figure 5). However, in *H. altissima*, although AMF colonization significantly increased photosynthetic rate in the RE30%, RE50%, and RE70% treatments, it did not improve iWUE in these treatments due to lack of strong reductions in *g*_s_ (Supplementary Figure S4). This result is consistent with the lack of significant differences in *g*_s_ detected between mycorrhizal and non-mycorrhizal plants in previous studies (Augé, 2000; He et al., 2017).

Drought stress impedes plant growth through peroxidative damage (Porcel and Ruiz-Lozano, 2004; Abogadallah, 2011; Hasanuzzaman et al., 2013); however, AMF can effectively reduce oxidative stress by increasing antioxidant enzyme activities in host plants (Benhiba et al., 2015; Mirshad and Puthur, 2016). The level of MDA is an indicator of the extent to which a plant has been exposed to peroxidative damage caused by drought stress (Uzilday et al., 2012). In the present work, AMF colonization significantly reduced MDA content in *L. chinensis* under the RE30% and RE50% treatments (Supplementary Figure S5A). Interestingly, AMF colonization induced a simultaneous reduction in MDA content and increases in CAT and SOD activities in *L. chinensis* under all rainfall exclusion conditions (Supplementary Figures S5, S6). In those cases where AMF colonization promoted CAT activity but not SOD activity, no significant AMF-induced reductions in MDA content were observed. However, where SOD activity was increased, MDA content was significantly reduced in the AMF-colonized plants. These results indicate that the mycorrhizal symbionts reduced peroxidative damage in *L. chinensis* by promoting CAT and SOD activity, with SOD activity playing a decisive role. We cannot rule out potential synergistic effects between SOD activity and CAT activity in *L. chinensis*. Similar effects of AMF on SOD and CAT activity under abiotic stress have been reported previously (Xun et al., 2015; Pedranzani et al., 2016; Chang et al., 2018). In contrast, in *H. altissima*, although AMF colonization enhanced the activities of CAT alone or of both CAT and SOD, no difference in MDA content was observed between the static and rotation treatments. In summary, these results suggest that the effects of AMF on plant photosynthesis, iWUE, and antioxidant enzyme activities vary with the magnitude of rainfall exclusion and between plant functional groups. Moreover, glutathione reductase (GR) and ascorbate peroxidase (APX) are important antioxidant enzymes for scavenging ROS in plants and should be studied in future work.

### Functional Group Differences in the Contribution of Arbuscular Mycorrhizal Fungi to Drought Resistance

Our results indicated that the physiological response to water stress differed between *L. chinensis* and *H. altissima*. More interestingly, we found that AMF play an important and efficient role in the resistance to water stresses in *L. chinensis* (C_3_ grass), compared to *H. altissima* (C_4_ grass). The symbiotic association between AMF and plants is one of most important factors contributing to plant growth under water stress (López-Ráez, 2016). Several studies have illustrated that arbuscular mycorrhizal symbiosis assists plants in alleviating drought stress by multiple mechanisms compared to non-mycorrhizal plants (Ruiz-Lozano et al., 2016; Zhang et al., 2018). The present study confirms previous findings by analyzing the effects of mycorrhizal associations on plant photosynthetic characteristics and antioxidant enzyme activities. C_4_ plants evolved from C_3_ plants, and plants of the two functional groups differ both structurally and functionally in the responses to various aridity stresses (Nayyar, 2003; Nayyar and Gupta, 2006). Previous reports indicated that C_3_ plants are more suited to growth in temperate environments, whereas C_4_ plants evolved under tropical and arid conditions (Ward et al., 1999). C_4_ plants show greater resistance to drought stress than do C_3_ plants because they have superior water and nitrogen use efficiencies and greater adaptability to water deficit (Nelson et al., 2004; Worchel et al., 2013; Gundel et al., 2016). Therefore, it is possible that under water-limited conditions, the mycorrhizal contributions to stress tolerance are lower for C_4_ species than for C_3_ species; however, experimental studies on this topic are lacking. In the current research, the intensity of mycorrhizal colonization was markedly higher for the C_3_ species than for the C_4_ species in all of the rainfall exclusion treatments, suggesting that the mycorrhizal dependence of the C_3_ species was stronger than that of the C_4_ species. Differences in the species composition of AMF mycorrhizal communities have been observed between C_3_ and C_4_ plants (Lanfranco et al., 2018), and differences among AMF species in the benefits they provide have been observed (Kiers et al., 2011). Therefore, the contribution of mycorrhizae to drought resistance in the host plants was expressed as MR in this study (Chandrasekaran et al., 2016; Bowles et al., 2018). The results suggested that under the RE30% and RE50% treatments, the contribution of mycorrhizae to plant biomass was significantly greater in the C_3_ species than in the C_4_ species (Figure 4). The contributions of mycorrhizae to *A* and iWUE had similar trends to that of biomass promotion under all of the rainfall exclusion treatments (Figure 5). These results indicate that the productivity improvement in the C_3_ plant was mainly due to the enhancements of *A* and iWUE by AMF pathway under the rainfall exclusion conditions. However, no significant contribution of mycorrhizae to *A*, iWUE, or biomass was observed in either the C_3_ or C_4_ plant under the 70% rainfall exclusion treatment (Figure 4, 5). This result might be explained by the fact that AMF can be powerful competitors with host plants, especially for C_3_ plants, under severe water restriction conditions. In addition, C_4_ plants have greater adaptability to water stress than do C_3_ plants (Ward et al., 1999); therefore, C_4_ plants may begin to use the AMF pathway to cope with water deficits under extreme drought conditions. The mycorrhizal colonization intensity of the C_4_ species was significantly enhanced under the RE70% treatment relative to the RE30% and RE50% treatments (Figure 3). Under the RE70% condition, the mycorrhizal contributions to *A*, iWUE, and biomass were slightly higher in the C_4_ species than in the C_3_ species (Figure 4, 5). Whether C_4_ plants are more dependent on mycorrhizae than are C_3_ species under extreme drought requires investigation in the future. No difference in mycorrhizal contribution to *g*_s_ in either species was detected under the RE30% and RE50% conditions (Figure 5). This result may be attributed to the fact that plants tend to minimize rates of evaporation at particular rates of assimilation (Augé et al., 2015), maximizing WUE in C_3_ species.

Another difference observed between the C_3_ and C_4_ species in this study was in the contribution of mycorrhizae to antioxidant activities, which suggests differences between these plant species in the level of dependence on AMF to cope with water stress. For example, the mycorrhizal contribution to CAT activity was significantly higher in the C_3_ species than in the C_4_ species in all of the rainfall exclusion treatments except the RE70% treatment. Under the RE50 and RE70% conditions, mycorrhizal promotion of SOD activity was greater in the C_3_ species than in the C_4_ species (Figure 6). CAT, a common peroxisome, plays a significant role in plant defense against drought-induced oxidative stress. Under drought conditions, CAT can reduce plant peroxidative damage by converting toxic hydrogen peroxide into water and oxygen (Willekens et al., 1997). The greater contribution of mycorrhizae to the activity of this enzyme in the C_3_ species than in the C_4_ species suggests a larger role of AMF in the C_3_ species in the removal of photorespiratory H_2_O_2_ produced during drought stress (Noctor et al., 2002). SOD can catalyze the dismutation of superoxide to stable H_2_O_2_ in the chloroplast, mitochondria, and cytosol to reduce oxidative damage (Blokhina et al., 2003). Compared to C_3_ plants, C_4_ plants have a superior capacity to counter oxidative stress under water-deficit conditions (Nayyar and Gupta, 2006; Uzilday et al., 2012). The mycorrhizal contributions to MDA content indicated that the presence of AMF prevented peroxidation damage in the C_3_ species under the RE30% treatment. However, the molecular mechanisms underlying the effects of AMF on photosynthesis and antioxidant enzyme activity and their potential differences between different photosynthetic pathways require study.

## Conclusion

Our results suggest that AMF can improve plant growth and reduce drought damage by increasing photosynthesis, iWUE, and antioxidant enzyme activities and reducing MDA content under light and moderate drought conditions in the Songnen grassland. However, under extreme drought, the mycorrhizal contribution to drought-stress reduction was not significant. The mycorrhizal contribution to drought resistance was much higher in the C_3_ species than in the C_4_ species. This finding highlights the fact that AMF play a vital role in determining plant community composition *via* increasing the relative abundance of C_3_ species and reducing that of C_4_ species under drought stress.

## Author Contributions

TZ, WS, and JL designed the experiments and wrote the manuscript. JL, BM, HC, XY, WS, SL, and AL performed the field and laboratory work. JL analyzed the data.

## Conflict of Interest Statement

The authors declare that the research was conducted in the absence of any commercial or financial relationships that could be construed as a potential conflict of interest.
