# Supplementary material for: Arbuscular Mycorrhizal Fungi Alleviate Drought Stress in C3 (Leymus chinensis) and C4 (Hemarthria altissima) Grasses via Altering Antioxidant Enzyme Activities and Photosynthesis
Source: Front Plant Sci. 2019 Apr 30;10:499. doi: 10.3389/fpls.2019.00499 (PMC6503820; doi:10.3389/fpls.2019.00499)
Supplement: Supplementary file 1 [file Data_Sheet_1.docx]

**Supplementary materials**

**Table S1** The contribution of mycorrhizal fungi (Static - Rotation) to the biomass, gas exchange and antioxidant metabolism in *L. chinensis* and *H. altissima*. Data are reported as mean ± 1 SE (n = 4). The values greater than zero represents positive effects of mycorrhizal to plant, and less than zero represents negative effects.

| Treatments | Species | Biomass  (g core^-1^) | *A*  (μmolCO_2_ m^-2^ s^-1^) | *g*_s_  (molH_2_O m^-2^ s^-1^) | iWUE  (μmolCO_2_ mol^-1^H_2_O) | MDA  (μmol g^-1^ d.wt) | CAT  (μmol min^-1^ mg^-1^ protein) | SOD  (U mg^-1^ protein) |
| --- | --- | --- | --- | --- | --- | --- | --- | --- |
| RE0% | *L. chinensis* | 0.33±0.07 | 2.30±0.7 | 0.08±0.02 | -2.67±0.5 | -0.29±0.3 | 0.016±0.03 | 1.71±0.1 |
|  | *H. altissima* | 0.49±0.4 | 1.78±1.0 | 0.03±0.02 | 0.93±0.2 | -1.09±0.3 | 0.001±0.05 | 0.57±0.1 |
| RE30% | *L. chinensis* | 0.46±0.06 | 3.05±0.5 | 0.05±0.01 | 4.67±0.6 | -1.93±0.07 | 0.017±0.05 | 3.25±0.5 |
|  | *H. altissima* | 0.55±0.1 | 1.39±0.2 | 0.05±0.01 | -2.28±0.3 | 0.43±0.5 | 0.005±0.01 | 2.33±1.0 |
| RE50% | *L. chinensis* | 0.45±0.06 | 3.84±0.9 | 0.07±0.01 | 6.73±0.5 | -1.52±0.6 | 0.002±0.01 | 8.03±0.5 |
|  | *H. altissima* | 0.62±0.1 | 2.57±0.3 | 0.04±0.01 | 1.22±0.7 | -0.28±0.7 | 0.007±0.01 | 4.48±0.7 |
| RE70% | *L. chinensis* | 0.32±0.07 | 0.16±0.3 | 0.01±0.01 | -2.47±0.3 | 0.40±0.2 | 0.005±0.01 | 3.19±0.2 |
|  | *H. altissima* | 0.38±0.06 | 0.65±0.1 | 0.01±0.01 | 3.73±0.6 | 1.16±0.5 | 0.002±0.03 | -5.4±0.7 |


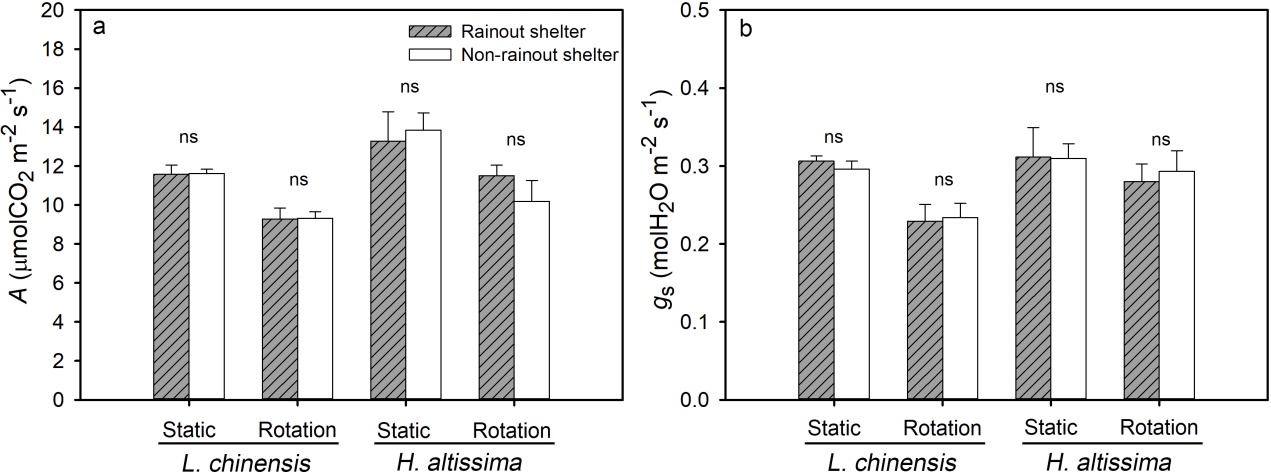


**Fig. S1** The difference in *A* (**a**) and *g*_s_ (**b**) between rainout shelter and non-rainout shelter. Data are reported as mean ± 1 SE (n = 4). ns represents no significant differences between the RE0% treatment and the control treatment.

**
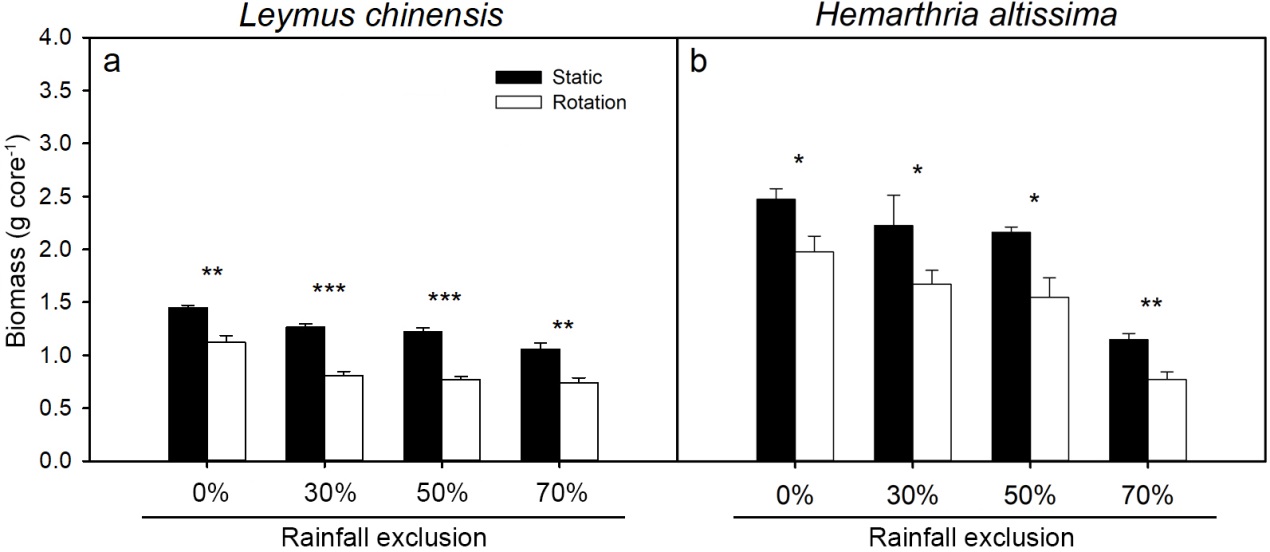
**

**Fig. S2** Effect of AMF on biomass in *L. chinensis* (**a**) and *H.* *altissima* (**b**) under different rainfall exclusions. Rotation treatment (white) represents the control without AMF, while static treatment (black) represents colonized with the AMF. Growth conditions were rainfall exclusion 0%, 30%, 50%, and 70%, respectively. Within each rainfall exclusion treatment, data are reported as mean ± 1 SE (n = 4), *asterisk* indicates significant differences: **P* < 0.05, ***P* < 0.01, ****P* < 0.001.


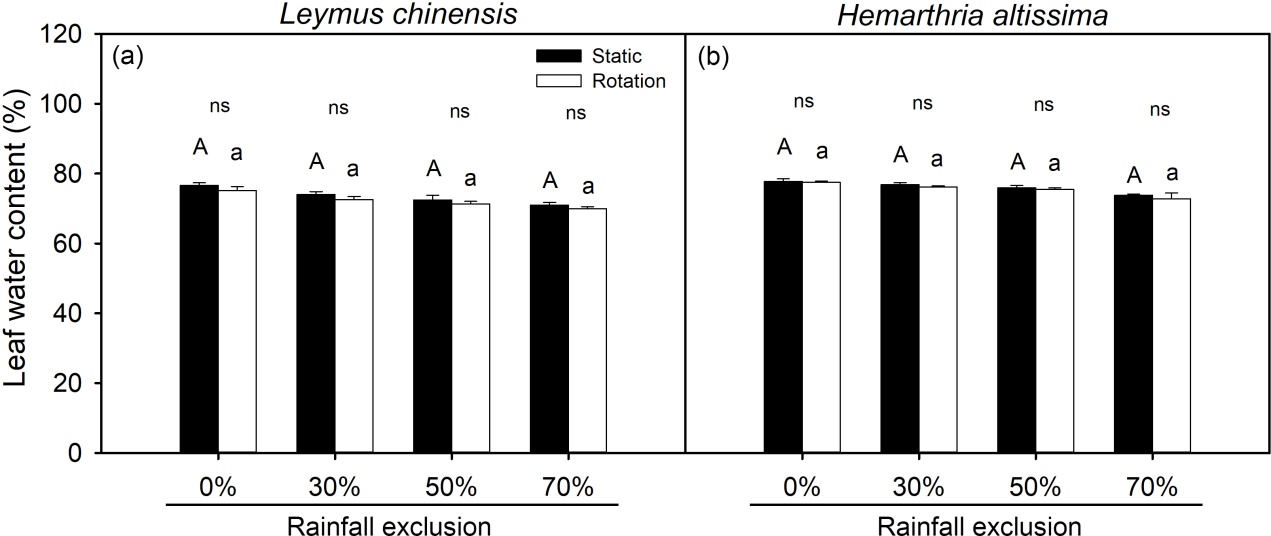


**Fig. S3** Leaf water content (%) in *L. chinensis* (**a**) and *H. altissima* (**b**). Different lowercase and capital letters indicate significant differences (*P* < 0.05) among the rainfall exclusion treatments with either the static treatment or the rotation treatment. Data are reported as mean ± 1 SE (n = 4), ns represents no significant differences between the rotation treatment and the static treatment.

**
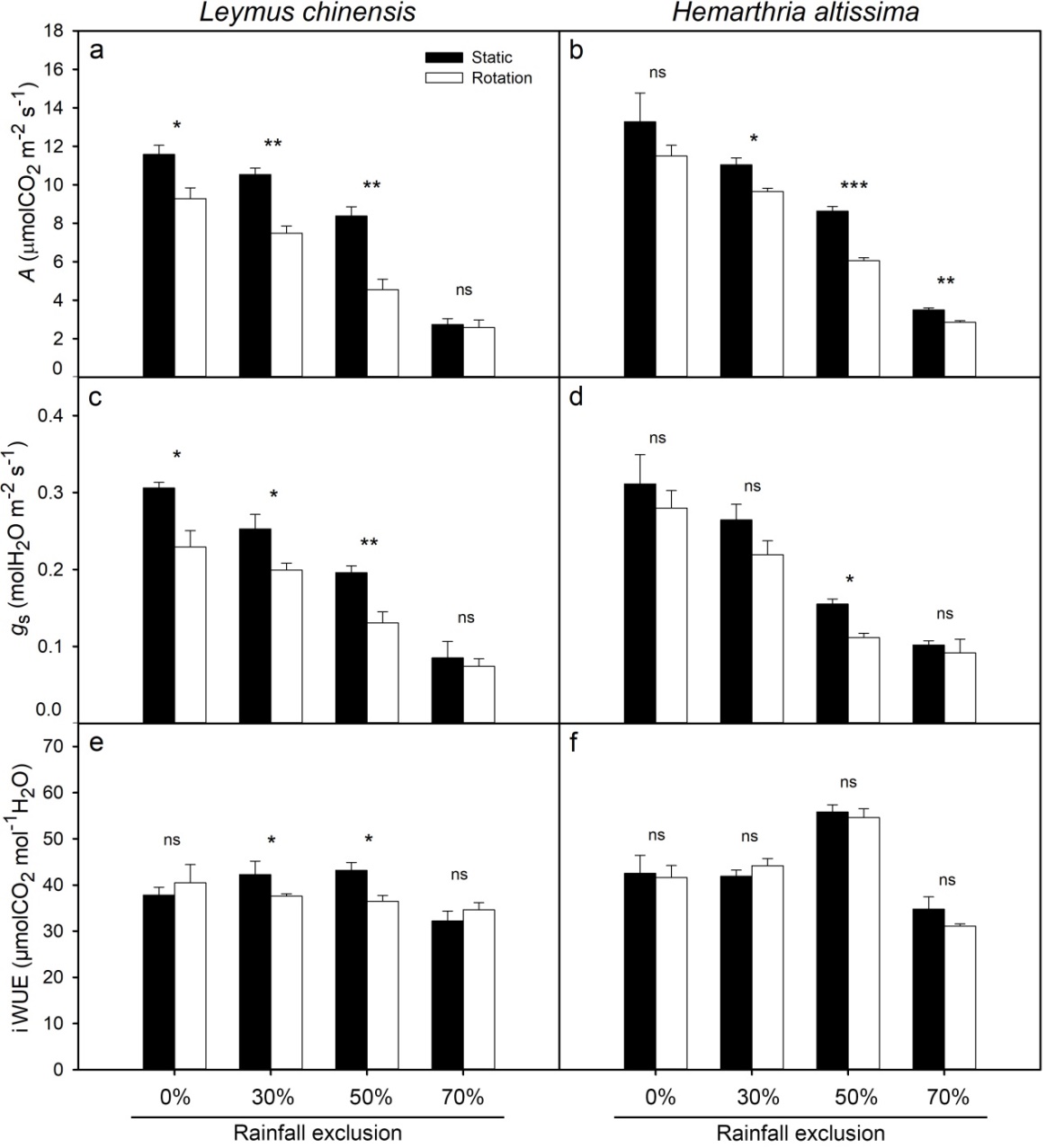
Fig. S4** Effect of AMF on photosynthesis characteristic in *L. chinensis* (**a**) and *H.* *altissima* (**b**) under different rainfall exclusions. Rotation treatment (white) represents the control without AMF, while static treatment (black) represents colonized with the AMF. Growth conditions were rainfall exclusion 0%, 30%, 50%, and 70%, respectively. Within each rainfall exclusion treatment, data are reported as mean ± 1 SE (n = 4), *asterisk* indicates significant differences: **P* < 0.05, ***P* < 0.01, ****P* < 0.001, and ns represents no significant differences between the rotation treatment and the static treatment.

**
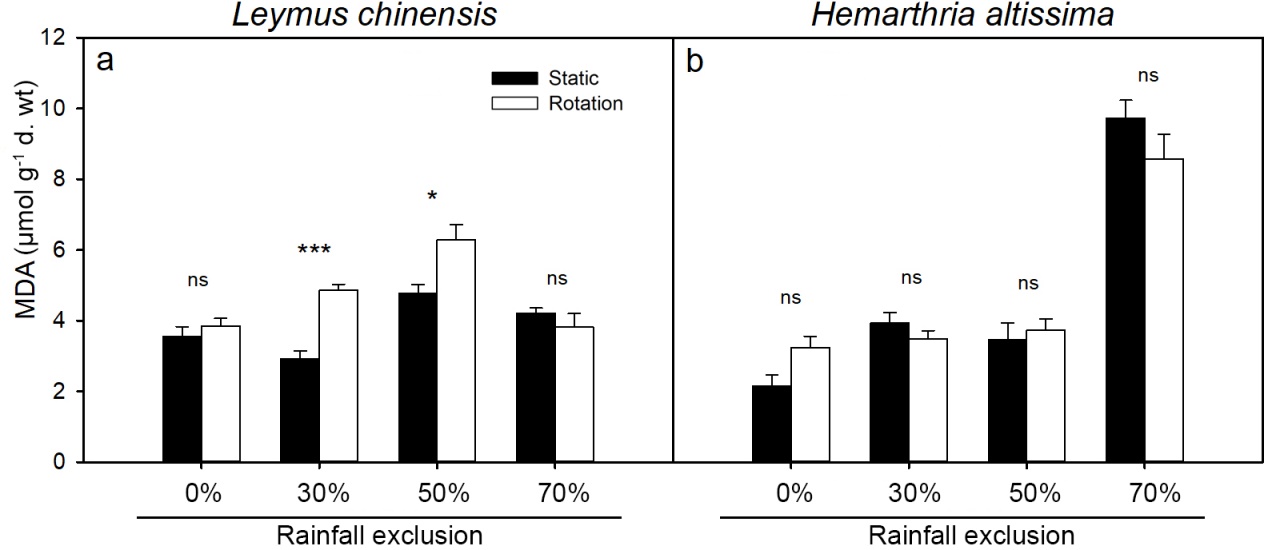
Fig. S5** Effect of AMF on contents of MDA in *L. chinensis* (**a**) and *H.* *altissima* (**b**) under different rainfall exclusions. Rotation treatment (white) represents the control without AMF, while static treatment (black) represents colonized with the AMF. Growth conditions were rainfall exclusion 0%, 30%, 50%, and 70%, respectively. Within each rainfall exclusion treatment, data are reported as mean ± 1 SE (n = 4), *asterisk* indicates significant differences: **P* < 0.05, ***P* < 0.01, ****P* < 0.001, and ns represents no significant differences between the rotation treatment and the static treatment. **
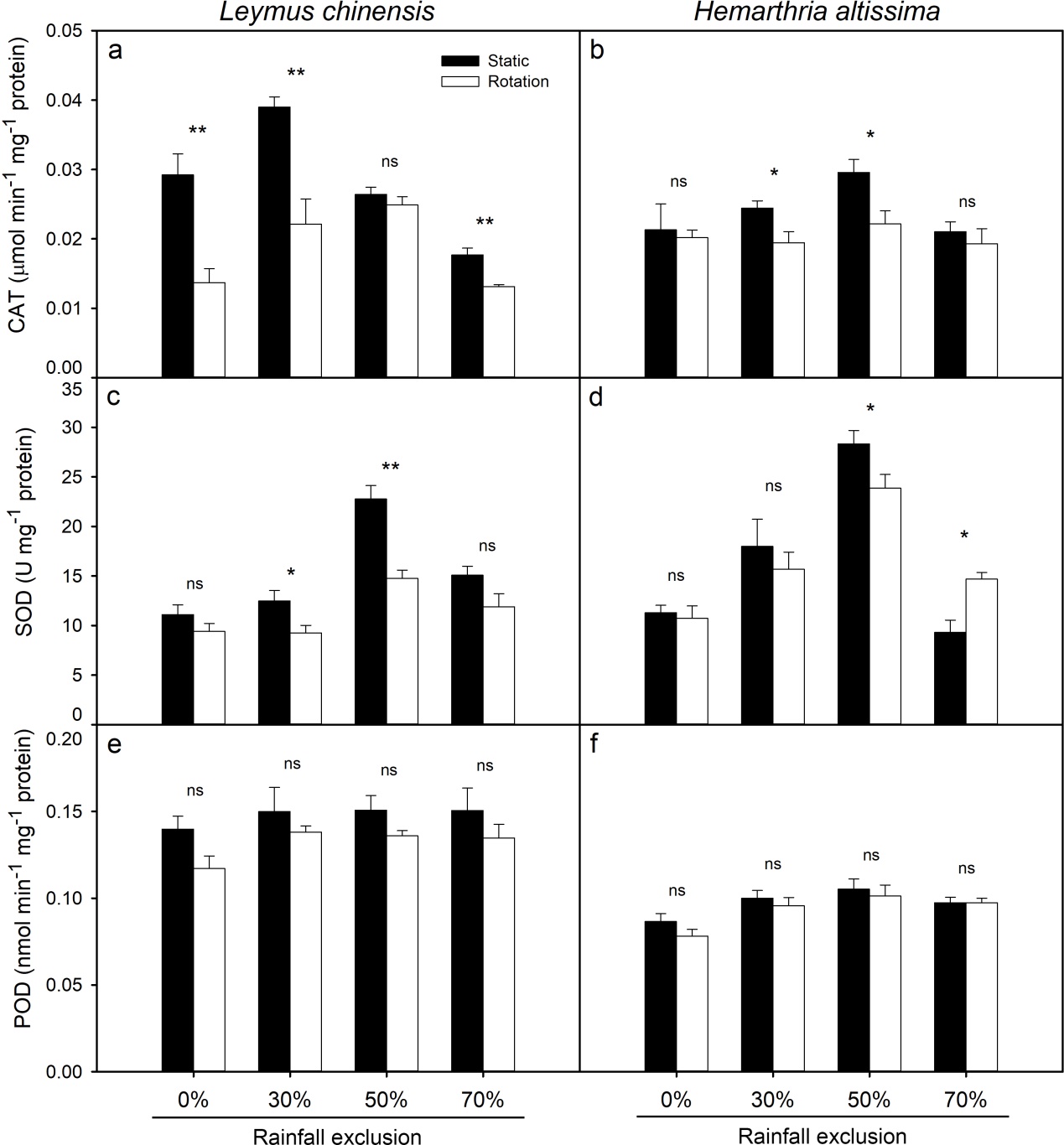
**

**Fig. S6** Effects of AMF on antioxidant enzyme activities in *L. chinensis* (**a, c, e**) and *H.* *altissima* (**b, d, f**) under different rainfall exclusions. Rotation treatment (white) represents the control without AMF, while static treatment (black) represents colonized with the AMF. Growth conditions were rainfall exclusion 0%, 30%, 50%, and 70%, respectively. Within each rainfall exclusion treatment, data are reported as mean ± 1 SE (n = 4), *asterisk* indicates significant differences: **P* < 0.05, ***P* < 0.01, and ns represents no significant differences between the rotation treatment and the static treatment.
